# Breaking Boundaries in Pneumonia Diagnostics: Transitioning from Tradition to Molecular Frontiers with Multiplex PCR

**DOI:** 10.3390/diagnostics14070752

**Published:** 2024-04-02

**Authors:** Alyssa M. Walker, Tristan T. Timbrook, Benjamin Hommel, Andrea M. Prinzi

**Affiliations:** 1 bioMerieux, 69280 Marcy L’etoile, Franceandrea.prinzi@biomerieux.com (A.M.P.); 2 Department of Pharmacotherapy, College of Pharmacy, University of Utah, Salt Lake City, UT 84112, USA

**Keywords:** infectious diseases, pneumonia, molecular diagnostics

## Abstract

The advent of rapid molecular microbiology testing has revolutionized infectious disease diagnostics and is now impacting pneumonia diagnosis and management. Molecular platforms offer highly multiplexed assays for diverse viral and bacterial detection, alongside antimicrobial resistance markers, providing the potential to significantly shape patient care. Despite the superiority in sensitivity and speed, debates continue regarding the clinical role of multiplex molecular testing, notably in comparison to standard methods and distinguishing colonization from infection. Recent guidelines endorse molecular pneumonia panels for enhanced sensitivity and rapidity, but implementation requires addressing methodological differences and ensuring clinical relevance. Diagnostic stewardship should be leveraged to optimize pneumonia testing, emphasizing pre- and post-analytical strategies. Collaboration between clinical microbiologists and bedside providers is essential in developing implementation strategies to maximize the clinical utility of multiplex molecular diagnostics in pneumonia. This narrative review explores these multifaceted issues, examining the current evidence on the clinical performance of multiplex molecular assays in pneumonia, and reflects on lessons learned from previous microbiological advances. Additionally, given the complexity of pneumonia and the sensitivity of molecular diagnostics, diagnostic stewardship is discussed within the context of current literature, including implementation strategies that consider pre-analytical and post-analytical modifications to optimize the clinical utility of advanced technologies like multiplex PCR.

## 1. Introduction

**Figure 1:**
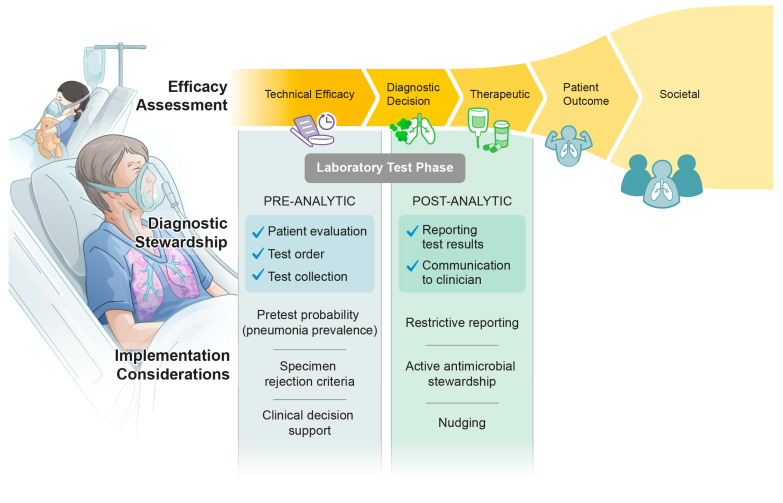
Diagnostic efficacy framework with implementation considerations.

Rapid molecular microbiology testing has become a cornerstone in infectious diseases, developing a significant presence in microbiology laboratories and shaping public health and guideline recommendations for antimicrobial stewardship (AMS), particularly in bloodstream infections [1,2]. This trend extends to pneumonia, where molecular platforms now offer sample-to-answer highly multiplexed PCR (mPCR) for comprehensive viral and bacterial etiologies, as well as antimicrobial resistance (AMR) markers and semi-quantitative assessments. The developing role of molecular testing in pneumonia diagnostics has been highlighted in recent literature establishing increased sensitivity and faster time to results compared to culture-based methods [3]. Despite the technical superiority of molecular testing, debates continue regarding the role of molecular testing in clinical practice. Important challenges and areas for future research include cost-effectiveness, differentiating between colonization and true infection, utility of semi-quantitative values from mPCR, and clinical impact. The evolving landscape of pneumonia testing yields substantial opportunities for clinical microbiologists and bedside providers to collaborate in developing implementation strategies to maximize the clinical utility of these advanced diagnostics in driving outcomes [4,5] (Figure 1). In this comprehensive review, we explore these complex and multifaceted issues, delving into the current evidence on clinical performance of mPCR pneumonia testing, guideline recommendations, lessons from previous advances in microbiology, and future directions for this advanced testing with diagnostic stewardship. The utility of alternate specimen types (outside of respiratory samples) will not be covered in this paper. Additionally, molecular technologies other than mPCR are out of scope for this review and will not be discussed.

## 2. Performance of mPCR vs. Culture

### 2.1. mPCR Discordance with Respiratory Culture

Pneumonia mPCR panels have demonstrated a significant advancement in their ability to detect pathogens not recovered by standard culture methods [6,7]. A landmark study in this space by Rand et al. evaluated the efficacy of a semi-quantitative mPCR panel compared to standard microbiology in hospitalized patients who underwent bronchoalveolar lavage (BAL) or endotracheal aspiration (ETA) as part of routine care, demonstrating high sensitivity (98.55%) but lower specificity (69%) for the mPCR compared to conventional microbiology [8]. The authors attributed the discrepancy in test specificity to the molecular panel’s ability to detect additional organisms not identified by culture-based methods. Notably, bacterial detections by the pneumonia (PN)panel showed a strong positive correlation with the white blood cell (WBC) count observed in the initial Gram stain, suggesting a reaction from the host, possibly in response to the identified bacteria.

In a study designed to understand the attributes of specimens with discordant mPCR detections, Rabin et al. examined the cellular characteristics of 65 culture-negative, PCR-positive BAL samples in mechanically ventilated patients with suspected pneumonia. They found that these samples exhibited markers indicative of infection with significantly higher BAL WBC count, neutrophil percentage, and amylase levels compared to culture-negative, PCR-negative samples [9]. To further explore the discrepancy between mPCR positivity and negative cultures, the investigators assessed how often repeat cultures became positive with the original organism identified by mPCR. An analysis of 17 culture-negative, PCR-positive samples revealed that upon repeat BAL, 35% were culture-positive for the organism identified by PCR in the original BAL sample. The authors suggested that these discordant cases (i.e., initial mPCR positivity and negative culture, followed by subsequent positive culture) may represent early infection. This study highlights the complexity of respiratory specimen timing, collection, and analysis in the critically ill patient population, factors that may significantly impact result interpretation for both mPCR and culture.

mPCR assays enable the reliable and simultaneous identification of various organisms that may not have been included in the differential diagnosis as well as those that are challenging to isolate via conventional methods. In particular, existing standard of care methods for virus detection rely on singleplex molecular assays limited to select viral pathogens [10,11], and viral culture is rarely performed due to slow turnaround time and the need for significant technical expertise and resources. A recent performance study of residual specimens reflected that only about one in four patients with a positive viral detection by mPCR had a clinician-ordered test for viral pathogens [10]. Moreover, detecting bacteria that require laborious methods or special growth media including elusive *Legionella* serotypes is simplified with molecular pneumonia assays [12]. The use of mPCR provides the opportunity to overcome many challenges with improved sensitivity and turnaround time, combined with comprehensive detection abilities that support clinicians in identifying a causative agent without relying on a hypothesis-driven approach [12].

### 2.2. Impact of Antimicrobial Exposure on Diagnostic Yield

A factor that must be considered in the evaluation of diagnostic test performance is the administration of antibiotics prior to specimen collection. The impact of antibiotics on organism growth has been demonstrated as early as one hour after IV antibiotic administration [13] and has been associated with a 50% decrease in culture yield [14]. Despite the known impact of antibiotic use on culture yield, studies comparing the performance of mPCR to standard culture demonstrated that 50–76% of patients with a positive mPCR test and negative culture received antibiotics before sample collection [8,10].

Fratoni et al. explored the hypothesis that molecular approaches might be less influenced by antibiotic exposure, leading to additional bacterial detections not identified by culture [15]. Their in vitro study compared an mPCR pneumonia panel with conventional culture methods using remnant BAL fluid exposed to clinically relevant antibiotic concentrations. Antibiotics were added to BAL samples containing standardized inoculums of various bacterial strains to simulate peak and trough pulmonary concentrations. The mPCR panel accurately identified all bacterial isolates at concentrations ≥ 10^6^ genetic copies/mL, regardless of antibiotic exposure. Conventional culture methods, however, showed decreased yields under antibiotic exposure, particularly for wild-type gram-negative bacteria. Of these gram-negative bacteria identified by mPCR, 86.3% did not grow or demonstrated insignificant growth in culture [15]. Alternatively, discordance between testing modalities was not observed for methicillin-sensitive and methicillin-resistant *Staphylococcus aureus* exposed to vancomycin. While this study used simulated conditions that may not fully replicate clinical complexities, it provides robust additional evidence that empiric antibiotic therapy may contribute to the differences in detections observed between molecular and conventional culture methods. These findings underscore the importance of considering antibiotic exposure when interpreting results from mPCR panels compared to cultures in clinical settings.

### 2.3. Quantification of Culture and mPCR Results

A significant challenge in clinical microbiology is the detection of oropharyngeal flora due to sample contamination introduced during collection from the respiratory tract. While this risk exists with traditional culture, it is increased with molecular testing due to improved sensitivity. The interpretation of microbiology test results from nonsterile sites is further complicated by the fact that bacterial burden and the presence of infection are not solely determined by a pathogen’s ability to cause harm but are also influenced by the effectiveness of the host’s immune system in mounting a defense [16]. Virulence, viewed as a dynamic outcome shaped by the interaction between the pathogen’s characteristics and the host’s immune response, underscores the complex nature of host–pathogen relationships. Evidence demonstrating associations between the inflammatory host response and bacterial load measured by molecular methods suggests that there is value in quantified pathogen detection [17,18,19]. Quantifying pathogens in respiratory samples is a technique some clinical laboratories may use to help discern whether contamination, colonization, or infection is present. For ventilator-associated pneumonia (VAP), major guidelines recommend quantitative or semi-quantitative culture methods, including thresholds for different sample types and with decreased quantities as sample volume increases [20,21].

While conventional culture-based methods remain fundamental in clinical microbiology, there is growing interest in exploring situations where molecular semi-quantification could offer advantages in clinical practice. However, before molecular semi-quantification can be fully embraced clinically, comparisons with semi-quantitative culture methods are necessary. A study by Murphy et al. highlighted the inherent differences between molecular and culture semi-quantification methods, indicating that the comparisons between CFU/mL and DNA copies/mL show no strict correlation [22]. Additionally, a comprehensive study by Ginocchio et al. reported higher DNA copies/mL in molecular semi-quantification compared to CFU/mL obtained from conventional culture methods [23]. In the study by Ginocchio et al., the molecular method demonstrated that values in sputum/ETA samples were, on average, 1 log higher, with 72% showing the same log or a 1 log increase. In BAL samples tested using the molecular method, values were approximately 1.5 log higher, with 62% showing a 1–2 log increase. Notably, 70% of molecular values were higher than culture values, 25% were similar, and 5% were lower. Buchan et al. found that all bacterial targets reported as ≥10^5^ CFU/mL in culture were reported as ≥10^5^ copies/mL by the molecular method [10]. Gastli et al. demonstrated that 90.1% of bacteria detected with ≥10^6^ DNA copies/mL grew at a significant level in culture [24]. Ferrer et al. established that 10^7^ copies/mL were clinically significant, correlating with ≥10^5^ CFU/mL in culture [25]. Posteraro et al. explored quantitative result agreement for 202 bacterial organisms identified in BAL and ETA samples, finding semi-quantitative agreement in 35.1% of cases [26]. In 56% of cases, culture values exceeded molecular values by less than 1 log10, while in 8.9% of cases, culture values exceeded the molecular method by more than 1 log10. Studies also suggest that the predominant pathogen detected by both methods is often the same, with an overall concordance of 93.2% [10]. These findings underscore the complexity of comparing semi-quantification of mPCR to culture.

The application of semi-quantitative results in clinical practice lacks consensus, partly due to challenges associated with establishing a threshold for distinguishing between colonization and infection. While some studies show promising correlations between semi-quantification of molecular and culture methods, discrepancies exist, emphasizing the need for further research to establish standardized protocols and guidelines for the clinical application of semi-quantification techniques. Additionally, the literature underscores the need for systematic reviews and meta-analyses to comprehensively assess the comparison between the two methods.

### 2.4. The Challenge of a Sub-Optimal Gold Standard

Respiratory culture has long been the standard of care test for diagnosing pneumonia, but its clinical utility is widely debated. Respiratory bacterial culture, particularly in patients receiving mechanical ventilation, suffers from poor specificity and sensitivity, slow turnaround time, and significant variability in processing and reporting [27,28]. Many causative agents of pneumonia, including atypical bacteria and viruses, may require specific growth conditions not met in routine cultures. As a result, potential pathogens may go undetected. These limitations make respiratory culture a poor gold standard for evaluating molecular diagnostic performance, as sensitivity and specificity assume a perfect reference method.

To evaluate the performance of a diagnostic test when the reference method is suboptimal, a statistical method known as Bayesian latent class analysis (BLCA) can be utilized [29]. BLCA overcomes sensitivity limitations by considering the imperfect nature of reference standards. It allows for the incorporation of multiple imperfect tests into an analysis. The term “latent class” refers to the unobservable or hidden classes, such as the true disease status of an individual. The BLCA approach does not require knowledge of the true disease status of a patient. It takes prior disease prevalence information and combines this with the diagnostic performance (e.g., sensitivity and specificity) of each test to estimate the probability of infection. 

A study which incorporated BLCA to better understand outcomes in the context of an imperfect comparator method was INHALE WP1, which evaluated the performance of two syndromic mPCR platforms in adult and pediatric patients with nosocomial pneumonia across 15 ICUs in the United Kingdom [7]. The initial performance analysis for each PCR target used routine microbiology and virology as the gold standard, which revealed that approximately half of the samples were fully concordant, and most of the remaining samples were partially concordant or had minor discordance due to additional detections by the mPCR panels. Notably, both mPCR assays frequently identified organisms that routine microbiology missed. The authors state that a subsequent BLCA was carried out due to concerns with culture being a substandard comparator and potentially leading to biased estimates of mPCR performance. The BLCA utilized models that did not assume which diagnostic method was ‘correct’ and revealed that routine microbiology was the least sensitive technique (27.1–68.7%) while sensitivity remained high for mPCR (83.9–99.3%). This study demonstrated via BLCA that molecular-based diagnostics outperform conventional microbiology in providing an etiologic diagnosis for patients with suspected hospital acquired pneumonia (HAP) and VAP. These findings suggest that culture is not an appropriate gold standard indicating a need for a new benchmark to measure the performance of molecular diagnostics in pneumonia.

## 3. Clinical Utility of mPCR

### 3.1. Clinical Relevance of Additional mPCR Detections

The significance and clinical interpretation of additional mPCR detections not detected in culture has, until recently, remained unclear. Rand et al. conducted an investigation to examine the link between molecular pneumonia panel results and clinical characteristics of patients with suspected HAP and VAP [30]. To identify potential correlations between mPCR detections and various health indicators, the mPCR pneumonia panel and routine microbiology results were considered against various clinical parameters including but not limited to BAL leukocyte percentage, clinical pulmonary infection score (CPIS), radiologic findings, ICU length of stay, maximum FiO_2_, maximum temperature, procalcitonin, serum white blood cell count, and minimum pO_2_, all on the day of culture, as well as discharge coding for pneumonia. Statistically significant associations were found between mPCR bacterial copy number and peak temperature, discharge coding for pneumonia, and BAL polymorphonuclear cell (PMN) percentage. Statistically significant associations were not found between clinical variables and culture results. As demonstrated in their previous study, these results also identified a significant linear correlation between Gram stain WBC and mPCR panel positivity, whether there was culture growth or not. Additionally, when no pathogens were detected by mPCR, there was no significant relationship with Gram stain WBC. Furthermore, patients with CPIS scores ≥ 6 were more likely to have specimens with a pathogen detected by mPCR than those without, irrespective of culture positivity. These findings indicate that molecular detections and copy numbers correlate with clinical indicators of host response to infection, regardless of culture growth [30]. Taken together, findings from this and other studies [9] support the notion that positive respiratory PCR testing, in the absence of positive cultures, may hold clinical significance in hospitalized patients suspected of pneumonia, proposing clinical utility of molecular testing in appropriate patient populations. Most existing studies concentrate on the application of mPCR for pneumonia in ICU patients, while its utilization in other areas, such as general wards and surgical units, remains relatively underexplored. Research to evaluate its clinical effectiveness in alternate settings could be of interest for future studies.

### 3.2. Potential Impact on Prescribing Behavior and AMS

The rapid turnaround time of pneumonia mPCR assays paired with the detection of AMR resistance genes provides the opportunity for faster and more appropriate therapy [7]. Several studies have evaluated the potential impact of mPCR on AMS in patients with suspected pneumonia, revealing that the majority (70–80%) of patients would be eligible for an antimicrobial change [10,31], including the opportunity to de-escalate in 48% [10] and escalate in 13% whose empirical regimen did not cover the identified pathogen [31]. Studies assessing pneumonia mPCR’s impact on actual changes to antimicrobial therapy are limited and have not consistently demonstrated the changes seen in studies focused on theoretical outcomes, illustrating the difference between practice and potential. In a retrospective study of adult ICU patients with clinically diagnosed pneumonia, Miller et al. did not find a statistically significant impact on antimicrobial use but did report a numerical reduction in the time to discontinuation of anti-MRSA (methicillin-resistant *Staphylococcus aureus*) agents and anti-pseudomonal therapy [32]. The authors noted that due to the onset of the pandemic, implementation and education for the pneumonia panel did not occur, and notably, the AMS intervention rate was low (~27%) [32].

Comparatively, in a study of ICU patients with pneumonia whose test results were paired with active AMS guidance, Poole et al. demonstrated that 80% of patients with mPCR testing received results-directed therapy compared to 29% of patients in the control group, and the time to results-directed therapy was significantly shorter in the mPCR group (2.1 h vs. 46.1 h) [33]. Additionally, there was a significant difference between groups concerning antibiotic de-escalation; 42% of patients in the mPCR group had antibiotics de-escalated compared to only 8% in the standard of care group, with de-escalation taking 41 h less in patients with samples tested using mPCR [33]. The authors concluded that mPCR testing improved antimicrobial use and appeared safe in their cohort of patients with pneumonia.

### 3.3. Molecular Diagnostics for Pneumonia in Special Populations

The advantages of using molecular diagnostics for pneumonia may be uniquely demonstrated within special populations, such as those with hematologic malignancies, solid organ transplants, or immunosuppressive diseases such as HIV. Often, patients with compromised immune systems are at increased risk of developing infections with atypical organisms that are difficult to grow in culture or identify using conventional test methods. A recent summary of findings from a consensus conference sponsored by the American Society of Transplantation notes that lower respiratory tract molecular assays may be useful in patients with focal consolidations, nodules, or cavities on chest imaging and when conventional microbiology testing is negative [34]. Additionally, the report states that molecular respiratory assays may be advantageous for solid organ transplant patients who have diffuse infiltrates on chest imaging since multiplex panels can detect many of the viruses associated with this chest-imaging pattern and for which there is treatment [34].

In recent years, research has demonstrated the improved performance of molecular testing compared to conventional methods for the diagnosis of *Pneumocystis* pneumonia [35,36,37]. While cases of *Pneumocystis* pneumonia in patients living with HIV/AIDS have decreased in recent years, rates of infection in transplant patients have increased. Patients with immunocompromising conditions, particularly those with hematologic malignancies, are at increased risk of severe disease and often have lower fungal loads than those with normal immune status [38]. In patients with reduced fungal loads, the performance of microscopy for detecting *Pneumocystis* may be significantly reduced [37]. Due to the increased sensitivity and high negative predictive value (NPV) of molecular testing for detecting *Pneumocystis* in respiratory samples, the European Conference on Infections in Leukemia (ECIL) recommends real-time PCR for the routine diagnosis of *Pneumocystis jiroveci* pneumonia (PJP) [38].

Additionally, positive *Pneumocystis* quantitative PCR (q-PCR) is included in the European Organization for Research and Treatment of Cancer–Mycoses Study Group Education and Research Consortium (EORTC-MSGERC) as evidence of probable disease [39]. The use of PCR to diagnose PJP has been contentious due to the challenges associated with differentiating between true disease and colonization, as well as uncertainty regarding the ubiquitous nature of *Pneumocystis.* However, studies have demonstrated the improved sensitivity of PCR in diagnosing *Pneumocystis* pneumonia and that immunocompromised patients with lower q-PCR values are usually associated with true infection and that colonization may be rare [36]. Furthermore, the Fungal PCR Initiative, a working group of the International Society for Human and Animal Mycology, continues to publish data that may support *Pneumocystis* test standardization and clinical adoption in the future [40]. Regardless, it is important to differentiate between colonization and infection across all patient groups, and molecular assays for pathogens like *Pneumocystis* should be paired with the pre-test probability, clinical data, guideline consultation, and other diagnostic test results to make an accurate diagnosis [35].

### 3.4. Cost Considerations

To date, a comprehensive evaluation on the cost-effectiveness of mPCR tests for pneumonia as a primary outcome has not been performed. However, a recent publication by Gilbert et al. estimated the costs associated with using an mPCR pneumonia test bundle compared to a standard-of-care test bundle, using historical cost data from their medical center [19]. Findings from that study demonstrated potential cost-savings with the mPCR test bundle, primarily due to no longer needing to perform certain standard of care tests, such as singleplex nasal PCRs or urinary antigen testing. Furthermore, in a prospective study of VAP patients that received mPCR, Guillotin et al. demonstrated that the cost to prevent one day of non-optimized antibiotics with mPCR was more expensive but more effective than relying on antimicrobial therapy recommendations alone [41]. This evidence points toward the value of mPCR in enhancing patient care while potentially streamlining healthcare expenditures.

## 4. Guideline Changes, Lessons, and Future Research

Recent research demonstrating increased insensitivity of mPCR pneumonia assays has resulted in several practice guidelines and society documents updating recommendations to endorse their use [42,43,44,45]. The 2023 ERS/ESICM/ESCMID/ALAT Guidelines for the Management of Severe community-acquired pneumonia (sCAP; severe CAP requiring admission to the ICU) indicate that a sputum or ETA sample should be collected for mPCR testing if it is available, whenever non-standard sCAP antibiotics are prescribed or considered. However, it is worth noting that empiric therapy selection using systematic risk assessment from validated risk scores is limited to two studies. Therefore, based on these recommendations, panel utilization may not be optimal [42], as the guideline authors suggest that a major benefit of mPCR testing would be an escalation of therapy for antibiotic-resistant pathogens [42]. Regarding the assessment of outcomes, the authors perceptively indicate that given the limitations of culture comparisons, only clinical data on outcomes can determine the true impact of management based on PCR results. Further, they highlight that optimal implementation will require rapid notification of results to prescribing physicians, and research around post-analytical clinical decision-making should be explored. Similarly to the ERS/ESICM/ESCMID/ALAT recommendations, the CHEST Sepsis Resources Steering Committee developed considerations on “Rapid Diagnostics for Infectious Diseases in the ICU”, noting that while traditional culture is the gold standard, it has limited sensitivity and can take days to return results, specifically indicating that mPCR panels are associated with 60–75% detection rates vs. 44% for standard culture [45].

Specific to COVID-19 patients, two guidelines have endorsed mPCR testing citing improved sensitivity [43,44]. In 2022, the Guidelines for COVID-19 Laboratory Testing for Emergency Departments from the New Diagnostic Technology Team of the Taiwan Society of Emergency Medicine were published and recommended that for severely ill patients, physicians should consider mPCR testing due to its ability to improve early and precise antibiotic treatment for improving the outcome [43]. In the same year, the 7th Guidelines Recommendations for Evidence-based Antimicrobial agents use in Taiwan (GREAT) working group published the “Recommendations and guidelines for the diagnosis and management of Coronavirus Disease-19 (COVID-19) associated bacterial and fungal infections in Taiwan” [44]. They indicated if syndromic mPCR testing is accessible, it may be considered for critically ill COVID-19 patients due to its excellent sensitivity, high NPV, and significantly decreased turnaround time along with the ability to improve, streamline, discontinue, or avoid antimicrobial use. The authors indicate that BAL and ETAs should be specimen types used for mPCR (over nasopharyngeal swabs). Overall, while some pre-analytical and post-analytical considerations are given within the guideline and society documents, how to best implement and use these tests remains an unanswered question. Though thoughtful proposals on approaches for implementation have been described elsewhere, much research is needed to validate these proposed and other implementation considerations [46].

**Figure 2:**
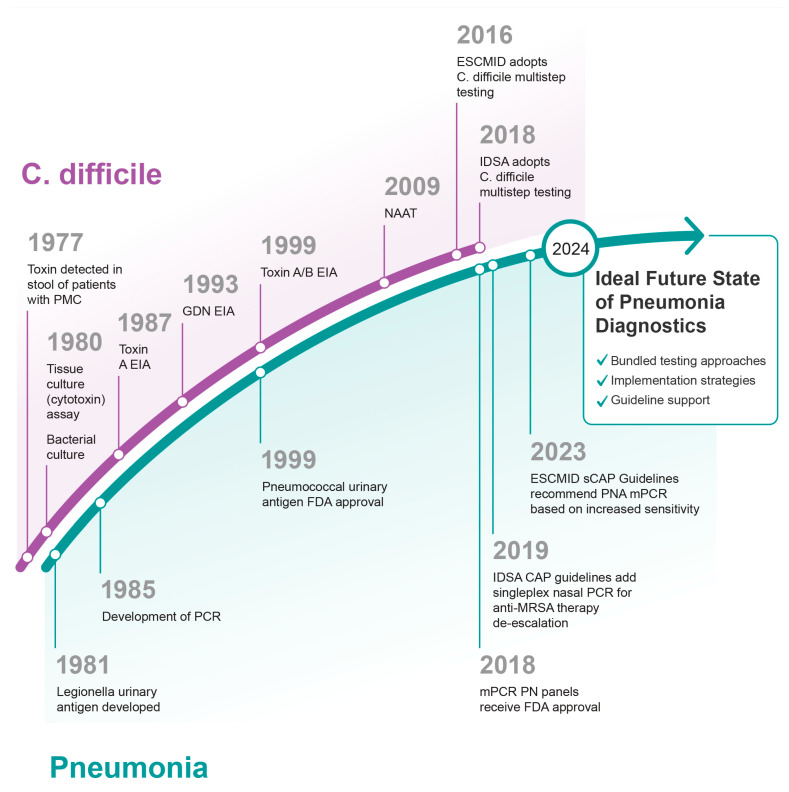
Progression of pneumonia diagnostics in relation to *C. difficile* diagnostics.

Given the increasing momentum of society recommendations on routine use of molecular mPCR testing for ICU patients related to its increased sensitivity and decreased turnaround time as compared to culture, how does one resolve issues with discordance to the gold standard of culture, particularly when not all detections are consistent with clinical disease? Analogous to the current paradigm shift in respiratory testing to a highly sensitive molecular test, *Clostridiodes difficile* testing has experienced a similar trajectory in advances in testing over the years which may offer critical insights into paths forward in implementing improved respiratory testing (Figure 2) [47].

Testing for toxin via tissue culture in suspected *C. difficile* infection (CDI) has been in practice since the 1980s, subsequently replaced by enzyme immunoassay (EIA) testing which while streamlining testing, has a low sensitivity. This led to the development of nucleic acid amplification tests (NAATs) which established highly sensitive testing. However, these highly sensitive tests are prone to detecting colonization as well, which occurs in 7–18% of hospitalized patients [48] and contributes to subsequent overdiagnosis [49]. Two landmark studies by Polage et al. and Planche et al. were conducted to address the issue of overdiagnosis by NAAT, which evaluated the natural history of this disease and the need for treatment in the setting of various toxin EIA and NAAT results [49,50]. These studies identified a correlation between cytotoxin production and clinical outcomes, indicating that this method best defines true cases of CDI. The data from these studies suggested that despite increased analytical diagnostic performance, NAAT is not always correlated to clinical disease. With this development, the question became: what guidance should be given on how to best use molecular *C. difficile* testing? Following the studies of Polage et al. and Planche et al., both the European Society of Clinical Microbiology and Infectious Diseases (ESCMID) and the Infectious Diseases Society of America (IDSA) updated their guideline recommendations on *C. difficile* testing [51,52]. In 2016, ESCMID released a multi-step algorithm within the *C. difficile* guideline update that combined a highly sensitive screening test (either NAAT or glutamate dehydrogenase [GDH] EIA) that could be reflexed to a highly specific test (toxin A/B EIA), thereby improving the clinical utility of testing. The following year, the IDSA released similar recommendations conditional on the use of a multistep algorithm. However, the IDSA guideline stipulated that NAAT alone could be used if facility-wide stewardship of testing was implemented. The shift in *C. difficile* testing recommendations provides a lesson for molecular testing in pneumonia. Implementation strategies (e.g., pre-analytical restrictions to clearly defined pneumonia criteria or the use of multi-step algorithms with biomarkers) may facilitate optimized clinical utility of pneumonia testing. 

Beyond the lessons gleaned from the *C. difficile* guidelines on pre-analytical approaches to improve the clinical utility of molecular testing, pneumonia guidelines offer additional insights into furthering mPCR implementation strategies [21]. In the IDSA 2016 clinical practice guidelines for the management of HAP and VAP in adults, the authors recommend holding rather than administering antibiotics for BAL results of <10^4^ CFU/mL growth. This recommendation is based on research from a retrospective study by Raman et al. evaluating the early (day of culture report) vs. late (any day after culture report) discontinuation of antibiotics in patients with culture-negative (<10 colony forming units/mL) quantitative bronchoscopy culture in VAP [53]. In an adjusted multivariable regression analysis, mortality was not statistically different between the two groups of early vs. late discontinuation (25.0% vs. 30.6%). Similar research with mPCR testing into the semi-quantitative results (copies/mL), therapy management, and other testing results’ correlations to clinical outcomes may better inform practice. 

In summary, the application of mPCR testing in clinical practice and guideline recommendations is rapidly evolving for the management of pneumonia. This pivotal paradigm shift has been driven by improved diagnostic performance (i.e., sensitivity) and decreased turnaround time of mPCR testing as compared to culture. This advancement in testing capability comes with the challenge of potentially over-diagnosing pneumonia, an issue that has occurred with other disease states using molecular testing. The lessons learned from the evolution of *C. difficle* testing offer invaluable insights for molecular testing in pneumonia, including the correlation of highly sensitive test results with clinical disease and the implementation of multi-step testing algorithms to enhance clinical utility. Future guidelines could benefit from incorporating these insights, potentially advocating for more refined criteria for test use, or integrating multi-step algorithms with biomarkers to balance the increased sensitivity of molecular tests against the risk of overdiagnosis. What lies ahead for pneumonia advanced diagnostic technologies hinges on the adoption of pre-analytical implementation strategies (i.e., diagnostic stewardship) as history has dictated its requirement to ensure the clinical utility of testing is optimized.

## 5. Current State of Diagnostic Stewardship and Future Directions

Diagnostic stewardship in infectious diseases, defined as modifying the ordering, testing, or reporting of results to improve patient care, has long been a cornerstone of medical laboratory testing and is now increasingly applied to most stages of clinical practice [54,55]. Furthermore, the relationship between diagnostic and AMS continues to be described, with both disciplines essential for optimal impact on the other [56]. Diagnostic decision-making and associated clinical behavior (e.g., antibiotic prescribing) are deeply rooted in behavioral determinants [57]. The decision to order a test is associated with the clinician’s baseline understanding of the pre-test probability of the disease of interest, and increasingly, with severe diseases such as VAP, the fear of missing something important [58,59,60,61].

However, studies assessing the accuracy of clinician estimates of disease probability before testing demonstrate that they cannot often perform appropriate Bayesian reasoning. For example, in a study of 553 clinicians across US outpatient clinics, the pre-test probability of disease was significantly overestimated for common infectious conditions, including urinary tract infections (UTIs) and pneumonia [59]. In a study of over 100 ICU clinical faculty and house staff, Soper and Albin demonstrated that provider estimates of VAP in ICU patients were consistently higher than evidence-based probabilities [58]. Consequently, overestimating the disease burden may lead to excessive diagnostic test use. The overuse of diagnostic tests is not benign. The suspected diagnosis of VAP is one of the primary indications for antibiotic use in the ICU, and increased use of respiratory culture has been associated with increased antibiotic use in ventilated patients [62]. A recent study by Vaughn et al. assessing the impact of AMS and diagnostic stewardship for urine cultures demonstrated that while restricted testing (i.e., diagnostic stewardship) was directly associated with decreased asymptomatic bacteriuria (ASB)-related antibiotic use, AMS was not [63]. These findings highlight the importance of diagnostic stewardship on clinical and AMS-focused outcomes and suggest that reducing testing in the pre-analytic period is impactful above and beyond AMS strategies.

Respiratory bacterial culture, although the standard of care, is significantly flawed and has become a target of stewardship interventions. Patients with pneumonia, particularly those in the ICU, are often complex and require care from a multidisciplinary team of clinicians who must make decisions rapidly [64]. While neither conventional culture nor rapid mPCR can fully differentiate between colonization and infection, diagnostic stewardship interventions in the pre-analytic period can help optimize testing without negatively impacting clinical outcomes. Using *C. difficile* as an example, pre-analytic interventions limiting unnecessary stool testing and subsequent false positives improved CDI diagnosis. Diagnostic stewardship algorithms for pneumonia testing could effectively be modeled after these protocols [65,66,67,68]. For example, Sick-Samuels et al. implemented a clinical decision support system (CDSS) for ETA culture testing on ventilated patients in the pediatric ICU. They noted a 41% monthly decrease in testing without changes in mortality, readmissions, or length of stay [64]. Albin et al. used a framework for developing complex health interventions, combined with an intervention design modeled after validated diagnostic stewardship intervention approaches for ASB, to create a diagnostic stewardship intervention for VAP [69]. This bundle included pre-analytic CDSS post-analytic result suppression and was associated with a 20% relative reduction in positive cultures with no negative impact on clinical outcomes. These pre-analytic considerations are particularly important in special populations like pediatrics, where the primary respiratory specimen collected is ETA due to children’s inability to produce sputum. Several studies have noted that repeat ETA specimens and specimens from patients with chronic tracheostomies rarely yield new information and may not be appropriate for mPCR or culture, highlighting the need for limiting unnecessary testing [70,71,72,73]. Respiratory testing is fundamental to the management of pneumonia, especially VAP, and these studies demonstrate opportunities for the use of diagnostic testing algorithms that may be extended to molecular testing and potentially support probabilistic decision-making in the pre-analytic period.

While less frequently explored, the opportunities for optimizing test use and antibiotic prescribing in the post-analytic period are abundant. As pre-test probabilities for pneumonia are grossly overestimated, so are likelihood estimates of disease after a “positive” respiratory culture [58]. Positive respiratory cultures, frequently contaminated with commensal microbiota, are often seen as synonymous with infection by treating clinicians. In patients receiving mechanical ventilation, reporting organisms from culture, regardless of significance (i.e., organism quantity, predominance in culture, or identification), is associated with increased and often unnecessary antibiotic use [62]. To help support judicious antimicrobial use and appropriate implementation of complex microbiological tests, “nudging” has been proposed as a promising post-analytic intervention strategy. Nudging may involve adding templated comments to microbiology reports that help with test interpretation, cascade reporting, or potentially result suppression. The nudging strategy supports optimal clinical decision-making while retaining clinician autonomy [74]. In addition to specimen screening in the pre-analytic and analytic periods, microbiologists and infectious diseases clinicians have a significant role to play in the post-analytic period. Involvement in building reporting algorithms, “nudges”, or providing clinical consultation and support of result interpretation are all effective post-analytic strategies that may help optimize the implementation and use of pneumonia mPCR.

In 2018, Musgrove et al. demonstrated the significant impact of a microbiology comment nudge for respiratory cultures [75]. By adding a comment to respiratory culture reports specifying whether or not MRSA or *Pseudomonas aeruginosa* were detected, the odds of antibiotic de-escalation increased nearly six-fold. Importantly, there were no differences in all-cause mortality between pre- and post-intervention groups. Alternatively, analytic results (e.g., Gram stain, cell counts, biomarkers) may be combined with guidelines or other available evidence to direct post-analytic reporting in a bundled approach [46]. In a pre-post study assessing the impact of a best practice alert for respiratory testing, Moradi et al. demonstrated a 2.2-day decrease in antibiotic days of therapy by using the electronic health record (EHR) to alert clinicians that their patient was positive for a virus by mPCR testing, and had a procalcitonin (PCT) result, suggesting the patient did not have a bacterial infection. This alert also notified the clinician that an antibiotic was prescribed, which was likely unnecessary based on the PCR and PCT test results [76]. As a part of the same VAP diagnostic stewardship bundle mentioned previously [58], the negative predictive value of the absence of neutrophils in BAL samples from patients with suspected VAP was harnessed. Culture results from BAL specimens with PMN cell percentages less than 50% were suppressed. Although the investigators noted a positive impact of the bundle on outcomes of interest, there was limited adoption of cell count testing and subsequent suppression of culture results. More research on barriers to post-analytic diagnostic stewardship adoption and the impact of bundled approaches should be explored.

The evidence on implementing rapid diagnostics shows that mPCR testing is most effective when combined with active AMS interventions rather than passive strategies [55,77,78]. The post-analytic period lends itself to strategies that focus on improving the communication of results, thereby improving clinical decision-making. Centers should consider using AMS-driven result review, communication with the treating team, and education framing of results to guide therapeutic interventions [46]. While advanced rapid diagnostic tests and stewardship interventions are crucial to improving patient care and demonstrating value, they often fall short of their full potential without using implementation frameworks or understanding barriers to adoption or drivers of behavior [55,57]. Adoption of molecular diagnostics for complex conditions like pneumonia may be initially limited due to increased sensitivity and the concern that it will lead to excessive antimicrobial use. This concern may be realized if consideration is not given to practice setting characteristics, education, barriers, facilitators, and other drivers of behavior change [79]. Variability in practice is immense concerning respiratory testing [27,28]. The importance of fully understanding the context of clinical settings that hope to implement rapid diagnostic testing cannot be overstated. This understanding can be further leveraged to develop context-specific stewardship interventions that optimize the use of more accurate and rapid tests by ensuring they are used in the right patient at the right time [80].

## 6. Conclusions

In conclusion, mPCR presents numerous advantages over traditional culture methods in diagnosing pneumonia. However, to fully harness its potential, a thoughtful approach to diagnostic stewardship across all testing periods and consideration of clinical and local practice contexts is imperative. Drawing insights from the evolution of testing for other infectious diseases, such as *C. difficile*, can inform our strategies moving forward. 

The ideal future of pneumonia diagnostics involves the integration of advanced technologies with great performance and rapid turnaround times. Bundled testing algorithms, complemented by stewardship-focused implementation approaches and robust follow-up protocols, may help achieve optimal use of this technology. By embracing these principles, it may be possible to improve patient care, streamline clinical decision-making, and mitigate the burden of pneumonia with greater efficacy and precision. 

## Data Availability

Not applicable.
